# Latent tuberculosis screening before kidney transplantation in the South of Brazil

**DOI:** 10.1590/2175-8239-JBN-2020-0189

**Published:** 2021-05-17

**Authors:** Gisele Meinerz, Cynthia Keitel da Silva, Damaris Mikaela Balin Dorsdt, Julia Bertoni Adames, Julio Pasquali Andrade, Pedro Enrico Ventura, Alexandre de Almeida Monteiro, Alessandro Comarú Pasqualotto, Valter Duro Garcia, Elizete Keitel

**Affiliations:** 1 Santa Casa de Misericórdia de Porto Alegre, Departamento de Nefrologia e Transplante de Rim e Pâncreas, Porto Alegre, RS, Brasil.; 2 Universidade Federal de Ciências da Saúde de Porto Alegre, Programa de Pós-Graduação em Patologia, Porto Alegre, RS, Brasil.; 3 Universidade Federal de Ciências da Saúde de Porto Alegre, Porto Alegre, RS, Brasil.; 4 Santa Casa de Misericórdia de Porto Alegre, Porto Alegre, RS, Brasil.

**Keywords:** Kidney Transplantation, Tuberculosis, Latent Tuberculosis, Interferon-gamma release assays, Tuberculin Test, Transplante de Rim, Tuberculose, Tuberculose Latente, Ensaios de liberação de interferon-gama, Teste Tuberculínico

## Abstract

**Background:**

Tuberculosis (TB) is a prevalent infection after kidney transplantation (KT) in high-burden countries. Latent tuberculosis infection (LTBI) screening includes previous TB history, chest radiograph findings, and tuberculin test (TST) and/or interferon-gamma release assays (IGRAs) results. We aimed to compare our routine LTBI screening of KT candidates and living donors (LD) with their IGRA results, and evaluate if this would improve isoniazid (INH) treatment referral.

**Methods:**

We evaluated adult KT candidates and LD with complete routine LTBI screening and QuantiFERON-TB® Gold In-Tube (QFT) testing. Blood samples were collected from April 4th, 2014 to October 31st, 2018, with follow-up until October 31st, 2019.

**Results:**

There were 116 KT recipients, with 30% QFT-positive results. Positive QFT was associated with past TB history (p=0.007), positive TST (p<0.0001), residual radiographic lesions (p=0.003), and diabetes (p=0.035). There were 25 LD, 40% had positive QFT. Positive QFT was associated with a positive TST (p=0.002). Positive QFT results increased INH referral in 80%. Post-transplant TB incidence was 2.6% in a median follow-up of 2 (1-33) months. No variables were associated with post-transplant TB. TB patients had inferior, although non-significant, 5-year graft survival (66.7% vs. 76.5%) (p = 0.402).

**Conclusion:**

In the present study, the association of QFT to our routine LTBI screening incremented INH treatment referral, but there was still a high incidence of post-transplant TB, possibly related to other forms of infection, such as new exposure and donor transmission.

## Resumo

**Histórico:**

Tuberculose (TB) é uma infecção relativamente comum pós-transplante renal (TR) em países com alta prevalência da doença. O rastreamento de infecção latente por tuberculose (ILTB) inclui histórico prévio de TB, achados de radiografia do tórax, resultados do teste tuberculínico (TT) e/ou de ensaio de liberação de interferon-gama (IGRAs). Nosso objetivo foi comparar nossa avaliação de rotina de candidatos ao TR e doadores vivos (DV) com seus resultados de IGRA, avaliando se aumentaria o encaminhamento para tratamento com isoniazida (INH).

**Métodos:**

Avaliamos candidatos adultos ao TR e DV com rastreamento para ILTB de rotina completo e coleta de testes QuantiFERON-TB® Gold In-Tube (QFT). Coletamos amostras sanguíneas de 4 de Abril, 2014 - 31 de Outubro, 2018, com acompanhamento até 31 de Outubro, 2019.

**Resultados:**

Avaliamos 116 receptores de TR, 30% sendo QFT-positivo. QFT positivo foi associado ao histórico prévio de TB (p=0,007), TT positivo (p<0,0001), lesões radiográficas residuais (p=0,003), diabetes (p=0,035). Avaliamos 25 DV, 40% apresentaram QFT positivo. QFT positivo foi associado a TT positivo (p=0,002). Resultados positivos do QFT aumentaram o encaminhamento para INH em 80%. A incidência de TB pós-transplante foi 2,6% em uma mediana de acompanhamento de 2 (1-33) meses. Nenhuma variável foi associada à TB pós-transplante. Pacientes com TB tiveram sobrevida do enxerto em 5 anos inferior, embora não-significativa (66,7% vs. 76,5%) (p = 0,402).

**Conclusão:**

Neste estudo, a associação do QFT à nossa avaliação de ILTB de rotina aumentou o encaminhamento para tratamento com INH, mas ainda houve alta incidência de TB pós-transplante, possivelmente relacionada a outras formas de infecção, como nova exposição e transmissão pelos doadores.

## Introduction

Tuberculosis (TB) is a prevalent and deadly infection after kidney transplantation, particularly in high-burden countries. Incidence rates are over 50-fold higher than in the general population^1^^-^ 6 and mortality rates range between 20 to 40%^1^^-^ 3 ^,^ 5 ^,^ 6.

Reactivation of latent foci is the most common source of infection^6^^-^ 9. The World Health Organization (WHO) estimates that one third of the global population have latent tuberculosis infection (LTBI)^10^. The risk of these individuals developing active disease is about 10-15% in their lifetime^11^, and it is even higher in immunosuppressed states^12^.

Thus, identifying kidney transplant (KT) recipients with higher risk of developing post-transplant TB allows timely interventions to try and prevent such ominous complications. There is no gold standard test to diagnose LTBI, so clinicians rely on surrogate methods to infer immune cellular response against the mycobacteria, including positive tuberculin skin test (TST) and/or interferon gamma-release assays (IGRAs).

Even though current international guidelines on KT candidates recommend using TST and/or IGRAs for LTBI screening^9^^,^ 12 ^-^ 15, IGRAs are more specific^16^ and have higher positive and negative predictive values^16^^,^ 17, but are more expensive^6^ and not as largely available as TST in developing countries.

Our hospital has one of the largest kidney transplant programs in Brazil, with over 4800 procedures performed since its implementation in 1977. Our routine evaluation of KT candidates and potential living donors (LD) includes screening for past history of TB, household contacts, TST results, and chest radiograph findings in asymptomatic subjects^2^^,^ 7 ^,^ 9 ^,^ 14. The limitations of this evaluation are low sensitivity and specificity, since (i) patients are not always reliable in recollecting lifetime exposure; (ii) TST lacks accuracy in chronic kidney disease patients due to decreased cellular response (anergy), (iii) there is a possibility of cross reaction in TST results with non-TB mycobacterial infection and BCG (bacilli Calmette-Guérin) vaccination^14^^,^ 18, and (iv) various studies demonstrated that a significant number of patients do not complete this screening, with a high proportion of missing TST results^3^^,^ 4 ^,^ 6 ^,^ 7.

In a previous study in our center, there was a high cumulative incidence of post-transplant TB (3.5% in 5 years) and a small number of LTBI diagnosis (12.4%)^5^. Almost all patients (98.6%) that received isoniazid (INH) therapy in the early post-transplant period did not develop active infection, reinforcing the efficacy of treating LTBI. One hypothesis was that our screening strategy was failing to detect patients that would benefit from LTBI treatment.

With that in mind, in this study we aimed to compare our current routine LTBI screening of KT candidates and LD (when applicable), with their IGRA results. We were particularly interested in evaluating if the strategy of screening with IGRA would improve INH treatment referral in a single center located in a country with high TB burden, and the possible impact in TB occurrence.

## Methods

### Study design and population

We conducted a prospective analysis of a cohort of KT performed at our center regarding latent tuberculosis infection screening and outcomes. We selected adult KT candidates and LD (when applicable) with complete routine LTBI screening that provided written informed consent to additional blood testing with QuantiFERON^®^-TB Gold In-Tube (QFT) (Qiagen, Hilden, Germany). We included patients that received KT from April 4^th^, 2014 to October 31^st^, 2018, with follow-up until October 31^st^, 2019. Exclusion criteria were individuals not willing to participate and recipients of conjugated transplants other than pancreas.

### Data collection

We collected data of KT recipients' age, gender, ethnicity, cause of kidney disease, comorbid conditions, immunosuppression, and LTBI. Donors' data on age, gender, ethnicity and living versus deceased donation were also collected.

### Clinical Ltbi Evaluation

All KT candidates and potential LD were evaluated at our outpatient clinic. Collected data included past TB history, TST results, and chest radiograph findings (apical fibronodular lesions, calcified solitary nodule, calcified lymph nodes, or pleural thickening). History and chest radiography are reassessed at transplant admission. Immunization against TB with BCG vaccination has been mandatory for children in Brazil since 1976.

### Tst screening

Tuberculin testing was conducted at the municipal health care facility and the results transcribed to the patients charts. TST was considered positive if ≥ 5mm for KT candidates and ≥ 10mm for LD^15^.

### Qft dssay

The blood samples were collected immediately before transplantation in the specific tubes, and all handling, incubation, and analysis were performed according to the manufacturer's instructions at the Hospital's Molecular Biology Laboratory. The results were available within 2 weeks post-transplant.

### Ltbi definition

KT candidates and LD were considered to have LTBI if any of the criteria described above were met and they were asymptomatic. Deceased donors' evaluation was based on information provided by the organ procurement organization, regarding past history of TB, incarceration, drug use, or relevant findings in the abdominal inspection during the kidney retrieval, such as lymphadenopathy and granulomas. Deceased donors with suspected or confirmed active tuberculosis are not considered for donation.

### Ltbi referral and treatment

LTBI treatment was conducted at the local primary care facilities, regulated by national guidelines. LTBI treatment is recommended after transplantation^19^ for recipients with a positive personal screening or with a donor's positive screening^15^. The recommended drug for LTBI treatment was INH 5-10mg/kg/day (maximum 300mg), to be initiated within 30 days of transplantation and maintained for 6 to 12 months, with the discretion of the local TB clinician (preferably 9 months). If clinical discharge was delayed for more than 30 days, treatment was initiated at the hospital, if clinically possible. Monitoring for liver toxicity was conducted at each clinical appointment, defined as an increase in aminotransferases above 3 times the normal reference value with symptoms (nausea, abdominal pain, jaundice) or above 5 times the normal reference value^19^.

### Tuberculosis definition

We defined active TB according to WHO criteria, with bacteriological confirmation by culture and/or polymerase chain reaction (PCR) test. Cases were classified as pulmonary when there was lung or tracheobronchial tree involvement, and extrapulmonary when affected exclusively other sites^20^. The date of confirmation of diagnosis was considered in the follow-up analysis.

### Tb treatment

TB treatment was conducted at the local primary care facilities. The recommended protocol was 2 months of rifampicin, isoniazid, pyrazinamide, and ethambutol (adjusted for creatinine clearance) followed by 4 months of rifampicin and isoniazid.

### Routine immunosuppression and post-transplant care

Induction therapy was tailored based on immunological risk, with depleting antibodies or interleukin-2 receptor inhibitors. Standard maintenance immunosuppression was triple therapy with a calcineurin inhibitor, an anti-proliferative drug, and low-dose steroids. For patients that needed TB treatment, calcineurin inhibitors' doses were adjusted to maintain target through levels according to post-transplant time, given three times daily while on rifampicin.

### Patients follow-up

All transplant patients were followed-up at our outpatient clinic, with medical consultations every week in the first three months, then biweekly until six months, monthly until one year, and then every two months. Follow-up started at transplantation date until the end of the study, death, or graft loss. Graft loss was defined as return to dialysis, new transplantation, or death with a functioning graft. All data on KT performed at our center are kept in an active database.

### Statistical analysis

Descriptive statistics were used to summarize the data. Quantitative variables are presented as medians and minimum-maximum values and compared using non-parametric tests. Qualitative variables are presented as numbers and percentages and compared using Fisher's exact test. All tests were 2-sided, and P values ≤0.05 were considered statistically significant. Independent variables with P values <0.2 in the univariate analyses were evaluated with multivariate logistic regression analysis. Graft and patient survival analyses were performed with Kaplan-Meier and significant values were determined by log-rank test. To estimate the magnitude of survival difference, we used Cox proportional hazards model, with 95% confidence interval (CI). Agreement between QFT and TST was calculated using kappa statistics and interpreted according to Landis and Koch: poor if k<0.20; fair if 0.21-0.40; moderate if 0.41-0.60; substantial if 0.61-0.80, and almost perfect if >0.81^21^. All analyses were performed using SPSS^®^ version 22 software.

The study was approved by the local Ethics Committee.

## Results

During the study period, there were 122 adult KT recipients with complete routine LTBI evaluation that also collected IGRA. There were 25 LD with complete routine LTBI evaluation and IGRA results. We excluded from the analysis 6 KT with indeterminate QFT results, yielding 116 patients. We excluded indeterminate results to better evaluate agreement between both tests.

Median follow-up was 36.5 (1-66) months.

### Kidney transplant recipients and ltbi screening

Characteristics of the 116 KT recipients are presented in Table 1. Median age was 47 (18-80) years; 65.5% were male, and 76.7% were Caucasian.

**Table 1:** Kidney transplant recipients's characteristics and risk factors for positive QuantiFERON-TB® Gold In-Tube Results

	Kidney recipients(n = 116)	Positive QFT(n = 35)	Negative QFT(n = 81)	Univariate (P)	Multivariate (P)	OR (95% CI)
Age, years (median, min-max)	47 (18-80)	54 (19-72)	44 (18-80)	0.057	0.66	-
Male (%)	76 (65.5)	26 (74.2)	50 (61.7)	0.21		
Caucasian (%)	89 (76.7)	26 (74.2)	63 (77.7)	0.61		
Diabetes (%)	22 (18.9)	11 (31.4)	11 (13.5)	**0.035**	**0.023**	**3.5** **(1.1 - 10.5)**
TB history (%)	4 (3.4)	4 (11.4)	0	**0.007**	0.99	**-**
TST result				**<0.0001**		
Positive (%)	21 (18.1)	14 (40)	7 (8.6)		**0.001**	**6.5** **(2.1 - 20.4)**
Negative (%)	95 (81.9)	21 (60)	74 (91.4)	* k = 0.354		
Abnormal chest radiograph (%)	7 (6)	6 (17.1)	1 (1.2)	**0.003**	0.99	-

Legend: QFT, QuantiFERON-TB® Gold In-Tube. TB, tuberculosis. TST, tuberculin skin test. k = kappa. CI, confidence interval.

There were 35 (30.2%) positive QFT results and 81 (69.8%) negative results. Positive QFT results were associated with past TB history (p = 0.007), positive TST (p < 0.0001), residual lesions on chest radiogram (p = 0.003), and preexisting diabetes (p = 0.035) in the univariate analysis. In multivariate logistic regression, positive TST (p = 0.001) and preexisting diabetes (p = 0.023) remained significantly associated.

There were 21 (18.1%) positive TST results (≥ 5mm). Agreement between TST and QFT results was fair, with k = 0.354. There was no variable significantly associated with test concordance.

### Living donors and ltbi screening

Characteristics of the 25 LD are presented in Table 2. Median age was 49 (33-66) years; 68% were female and 88% were Caucasian.

**Table 2:** Living Donors’s Characteristics And Risk Factors For Positive QuantiFERON-TB® Gold In-Tube Results

	Living Donor (n = 25)	Positive QFT (n = 10)	Negative QFT (n = 15)	p
Age, years (median, min-max)	49 (33-66)	48 (39-62)	51 (33-66)	0.85
Male (%)	8 (32)	4 (40)	4 (26)	0.66
Caucasian (%)	22 (88)	9 (90)	13 (86)	0.41
TB history (%)	0	0	0	n/a
TST result				
Positive (%)	6 (24)	6 (60)	0	**0.002**
Negative (%)	15 (60)	3 (30)	12 (80)	
Not done (%)	4 (16)	1 (10)	3 (20)	* k = 0.696
Abnormal chest radiograph (%)	1 (4)	1 (10)	0	0,40

Legend: QFT, QuantiFERON-TB® Gold In-Tube. TB, tuberculosis. TST, tuberculin skin test. k = kappa.

* performed with valid results from both tests.

No LD had previous history of TB, and one had residual lesions on the chest radiogram. No LD had diabetes. Four LD did not have TST performed due to temporary tuberculin distribution shortage.

There were 10 (40%) positive QFT results and 6 (24%) positive TST results (≥10 mm). Agreement between TST and QFT results was moderate (k= 0.696). The only variable associated with a positive QFT result was a positive TST in univariate (p = 0.002) and multivariate analysis (p = 0.039).

### Isoniazid referral and ltbi treatment

**Figure 1:**
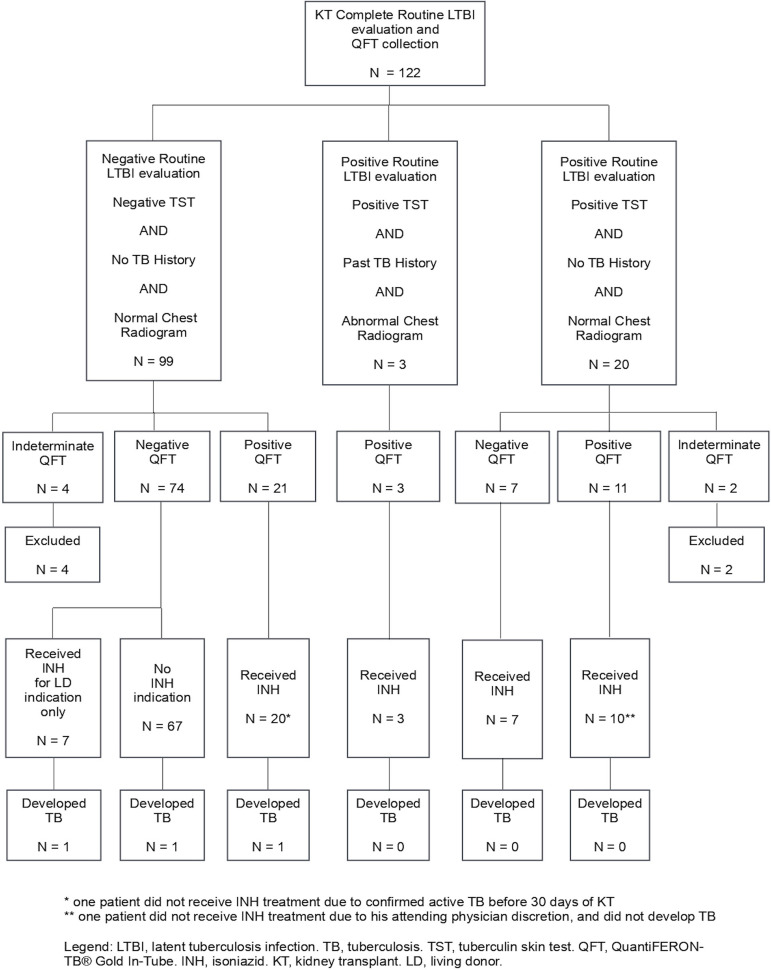
Flowchart demonstrating latent tuberculosis infection screening and treatment in kidney transplant recipients

There were 21 KT recipients with personal positive routine LTBI screening (14 with positive QFT), and 6 KT with LD's positive routine LTBI screening (all with positive QFT), totaling 27 patients. There were another 21 KT recipients with negative routine LTBI evaluation that had a positive QFT, plus one LD with positive QFT, totaling 22 patients considered positive for LTBI based on the QFT additional evaluation. In other words, the positive QFT results added 22 INH indications, totaling 49 referrals, an increment of 80%. This is summarized in Figure 1.

Forty-seven (95.9%) patients received INH for a minimum of 6 months. Only one (2.1%) patient developed hepatotoxicity after 6 months of INH therapy, with ALT elevation over 3x the reference values and abdominal pain, while treating CMV infection with intravenous ganciclovir - INH was suspended and abnormalities resolved.

Two patients did not receive LTBI treatment. One (2.2%) due to infectious complications and at his attending physician discretion: he did not develop tuberculosis during his follow-up (of 61 months). The other is described below.

### Post-transplant tb and impact on survival

There were 3 (2.6%) post-transplant tuberculosis cases during follow-up, in a median of 2 (1-33) months. Cumulative incidence was 3.2% in 5 years.

One patient with no history of TB, with a negative TST and a normal chest radiogram was asymptomatic at admission for transplantation. His positive QFT result was received within 2 weeks of transplantation. He developed pulmonary symptoms within 30 days of the procedure, before initiating INH. He presented cough and fever, and was diagnosed with positive acid-fast bacilli (AFB) and *M. tuberculosis* culture in bronchoalveolar lavage. He was cured, but lost graft function and returned to dialysis after 9 months of the diagnosis. The information regarding the deceased donor was negative for TB history and no abnormal findings were described during the removal of the organs. The other recipient did not develop TB.

One patient had negative personal LTBI screening with LD's positive TST and QFT. She received INH for 9 months. After two years, she developed disseminated tuberculosis with hepatosplenomegaly and lymphadenopathy, weight loss, and fever. Diagnosis was confirmed with positive AFB and culture in lung biopsy. She was cured with a functioning graft. There was no evidence of TB in close contacts at the time of active disease.

One patient with negative LTBI screening developed pulmonary TB within 3 months of transplantation. He presented dyspnea and fever, and was diagnosed with positive *M. tuberculosis* PCR in bronchoalveolar lavage. He received 6 months of treatment and was cured with a functioning graft. The recipient of the deceased donor's contra-lateral kidney developed kidney graft tuberculosis, diagnosed in the same period, but the patient was not part of this cohort. The information regarding the deceased donor was negative for TB history, and no abnormal findings were described during the removal of the organs.

All TB cases completed 6 months of the prescribed therapy (rifampicin, isoniazid, ethambutol, and pyrazinamide), adjusted for creatinine clearance, and none presented relevant toxicity. Calcineurin inhibitors doses were adjusted to maintain adequate through levels and monitored regularly. There was no multidrug-resistant TB case.

No variables were associated with post-transplant TB, as described in Table 3.

**Table 3:** Characteristics of kidney transplant recipients with and without post-transplant tuberculosis

	Kidney recipients (n=116)	Non-TB (n = 113)	T (n = 3)	p
Age, years				
(median, min-max)	47 (18-80)	47 (18-80)	52 (21-57)	0.71
Male (%)	76 (65.5)	74 (65.4)	2 (66.6)	0.96
Caucasian (%)	89 (76.7)	86 (76.1)	3 (100)	0.62
Diabetes (%)	22 (18.9)	22 (19.4)	0	0.39
TB history (%)	4 (3.4)	4 (3.5)	0	0.74
TST result				
Positive (%)	21 (18.1)	21 (18.5)	0	0.40
Negative (%)	95 (81.9)	92 (81.4)	3 (100)	
Abnormal chest radiograph (%)	7 (6)	7 (6.2)	0	0.65
QFT result				
Positive (%)	35 (30.2)	34 (30.0)	1 (33.3)	0,90
Negative (%)	81 (69.8)	79 (70.0)	2 (66.6)	
Living donor (%)	25 (21.5)	24 (21.2)	1 (33.3)	0,67
Living donor TST result				
Positive (%)	6 (24.0)	5 (20.8)	1 (100)	
Negative (%)	15 (60.0)	15 (62.5)	0	0,16
Unknown	4 (16.0)	4 (16.6)	0	
Living donor QFT result				
Positive (%)	10 (40.0)	9 (37.5)	1 (100)	0,40
Negative (%)	15 (60.0)	15 (62.5)	0	
Donor TB history	0	0	0	n/a
Donor age, years				
(median, min-max)	50 (1-74)	50 (1-74)	59 (44-65)	0,39
Donor female gender (%)	59 (50.9)	57 (50.4)	2 (66.6)	0,57
Induction therapy				
None (%)	7 (6)	7 (6.2)	0	0,85
Thymoglobulin (%)	46 (39.7)	45 (39.8)	1 (33.3)	
anti-IL2R (%)	63 (54.3)	61 (54.0)	2 (66.6)	
INH indication (%)	49 (43.3)	47 (41.6)	2 (66.6)	0.57
LTBI treatment (%)	47 (40.5)	46 (40.7)	1 (33.3)	0.77

Legend: TB, tuberculosis. TST, tuberculin skin test. QFT, QuantiFERON-TB ® Gold In-Tube. INH, isoniazid. LTBI, latent tuberculosis infection. anti-IL2R, anti-interleukin 2 receptor.

None of the TB patients died. TB patients had inferior, although non-significant, five-year graft survival (66.6% vs. 76.5%) and mean follow-up (46.3 ± 14.4 vs. 57.1 ± 1.8 months) (p = 0.402).

## Discussion

We described the results of a sample of adult KT recipients regarding LTBI evaluation with routine screening and additional IGRA testing. Our KT had 30% positive QFT and 18% positive TST results. The proportion of positive QFT results in KT recipients was similar to other reports^22^^-^ 24, and is in accordance with the WHO estimates on global LTBI prevalence^10^. Previous studies associated positive QFT results in chronic kidney disease patients with previous TB history and abnormal chest radiographs^25^. We also found these associations, as well as with diabetes and positive TST. Agreement between TST and QFT was fair (k = 0.354), similar to other studies in KT candidates^22^^,^ 23 ^,^ 26. Some authors found better agreement when considering TST ≥10mm^27^. One third of our positive TST patients had negative QFT results. The discrepancies between tests are explained by false-negative TST results due to cutaneous anergy, or false-positive TST results due to cross-reactivity with BCG vaccination or contact with non-tuberculosis mycobacteria^14^. It should also be noted that TST has a subjectivity aspect, and the tests were performed by different technicians, which could have impacted the reliability of their results. IGRAs are reported to be more sensitive and specific than TST^25^^,^ 27, without these confounding factors^28^.

Our LD had 40% positive QFT and 24% positive TST results. Agreement between the tests was moderate. There were no patients with positive TST and negative QFT results. In healthy individuals, cutaneous anergy is not expected, which could explain the difference from KT candidates. There was a high proportion of positive results, which could be expected because we are located in a high TB burden area.

One criticism regarding both tests is that they cannot differentiate latent from active TB. Current understanding is that latent and active TB are not two separate, opposite states, but the extreme manifestations of a continuum in the battle between pathogen and host, defined and marked by multiple factors, specially by host immune response^11^^,^ 28 ^-^ 30. In this study, one patient with positive QFT result developed active disease even before completing 30 days of the transplant procedure, although asymptomatic at admission and with a normal chest radiogram. He had a negative TST result at the time of waitlisting, so he would not be referred to INH with the routine screening. It is plausible that he had unrecognized active TB by transplantation. It could be argued that chest radiograms lack sensitivity to diagnose TB, and implementing thoracic computed tomography could improve the evaluation, as demonstrated in some populations^31^.

Associating QFT results to our routine screening incremented LTBI treatment referrals in 80%. There was a low incidence of hepatotoxicity with INH treatment (2.1%). Reports on INH efficacy in preventing TB demonstrate that high risk individuals that received LTBI treatment did not develop active disease^32^, and up to 22% of those that did not receive it developed TB^7^. Two recent meta-analyses on LTBI treatment demonstrated a significant relative risk reduction of 0.3 in developing TB^33^, and one recommended treatment for all KT recipients in endemic regions during the first year after transplantation^34^. Over 95% of our patients with INH indication received the medication, and only one (2.1%) developed active TB, two years after treatment. From the two patients that had INH indication but did not receive it, one did not develop TB during follow-up (over 60 months), and one developed respiratory symptoms before initiating treatment, as discussed above.

Regarding the timing of LTBI treatment, international guidelines recommend that KT candidates receive therapy ideally before transplantation, for a minimum of 9 months^2^^,^ 9 ^,^ 14 ^,^ 15, but it is not a consensus^6^^,^ 33. Our policy is to treat patients after active immunosuppression, for a minimum of 6 and preferably for 9 months. The patient that developed TB 2 years after INH treatment did not have positive personal LTBI screening, but her LD had. We hypothesized that new exposure is possible since we are located in a high burden area, but it could also be argued that the protective effect of INH does not last for longer periods. In this sense, treating LTBI before transplantation in patients with expected long waitlisting time may have a waning effect and require retreatment. A recent review also described a large number of centers that prescribe INH after transplantation^35^. Although the potential of more severe drug interactions is greater after transplantation, patients are monitored more closely in the initial months, which could improve the safety of this approach. Also, the risk of reactivation is greater after immunosuppression is commenced^36^.

Cumulative post-transplant TB incidence was 3.2% in 5 years, similar to our previous report^5^. It should be noted that this cohort is much smaller than the previous one and that they were not concurrent, so not directly comparable. Also, since proportionally more patients were referred to INH treatment, we cannot measure how many cases could have been prevented. Reported TB incidence is intimately related to local burden^3^^,^ 6, and in Brazil it ranges from 1.7 to 5.6%^37^^-^ 41.

We could also argue that LTBI treatment alone would not impact the incidence in high burden TB areas, where new exposure and donor transmission are also likely to occur. For these situations, other strategies could be discussed, such as reevaluating KT recipients over time for LTBI, more aggressive screening of deceased donors, or even universal treatment as some authors recommend^33^.

We did not find any factors associated with active TB development, probably because of the small number of cases.

There were no TB related deaths, and one third of TB patients lost graft function. Mortality has been reported from 6^4^ to 30%^5^^-^ 7, and graft losses around 25%^2^^,^ 5 ^,^ 7.

In conclusion, TB is a major complication after KT, especially in high burden countries like Brazil. Detecting individuals at higher risk of developing post-transplant TB and referring them to receive preventive treatment has been advocated by international guidelines as a mean to reduce its incidence and morbidity, but the best strategy to identify these individuals is not well established. The implementation of IGRAs as a screening tool for latent TB infection is meant to add sensitivity and specificity, but, as TST, it lacks capacity to discriminate from active disease. Also, the cost is considerably high to implement on a large scale. In the present study, associating QFT tests to the routine LTBI screening incremented treatment referral in 80%, and treatment was effective in preventing TB in all but one patient. There was still a high incidence of post-transplant TB, possibly related to other forms of infection, such as new exposure and donor transmission. It is important to conduct studies to analyze the financial impact of implementing IGRA as a large scale screening tool, versus the cost and risks of universal treatment for LTBI versus the burden of active TB and its impact on graft survival.
